# Biosynthesis Strategy of Gold Nanoparticles and Biofabrication of a Novel Amoxicillin Gold Nanodrug to Overcome the Resistance of Multidrug-Resistant Bacterial Pathogens MRSA and *E. coli*

**DOI:** 10.3390/biomimetics8060452

**Published:** 2023-09-25

**Authors:** Eman M. S. Halawani, Seham S. S. Alzahrani, Sanaa M. F. Gad El-Rab

**Affiliations:** 1 Department of Biology, College of Science, Taif University, P.O. Box 11099, Taif 21944, Saudi Arabia; halawani@tu.edu.sa; 2 Department of Biotechnology, College of Science, Taif University, P.O. Box 11099, Taif 21944, Saudi Arabia; 3 Botany and Microbiology Department, Faculty of Science, Assiut University, Assiut 71516, Egypt

**Keywords:** antimicrobial activity, amoxicillin gold nanodrug, MRSA, *E. coli*, multidrug resistance

## Abstract

The prevalence of multidrug-resistant (MDR) bacteria has recently increased dramatically, seriously endangering human health. Herein, amoxicillin (Amoxi)-conjugated gold nanoparticles (AuNPs) were created as a novel drug delivery system to overcome MDR bacteria. MDR bacteria were isolated from a variety of infection sources. Phenotype, biotype, and 16S rRNA gene analyses were used for isolate identification. Additionally, *Juniperus excelsa* was used for the production of AuNPs. The conjugation of AuNPs with Amoxi using sodium tri-polyphosphate (TPP) as a linker to produce Amoxi-TPP-AuNPs was studied. The AuNP and Amoxi-TPP-AuNP diameters ranged from 15.99 to 24.71 nm, with spherical and hexagonal shapes. A total of 83% of amoxicillin was released from Amoxi-TPP-AuNPs after 12 h, and after 3 days, 90% of the medication was released. The Amoxi-TPP-AuNPs exhibited superior antibacterial effectiveness against MRSA and MDR *E. coli* strains. Amoxi-TPP-AuNPs had MICs of 3.6–8 µg mL^−1^ against the tested bacteria. This is 37.5–83 fold higher compared to values reported in the literature. Amoxi-TPP-AuNPs exhibit a remarkable ability against MRSA and *E. coli* strains. These results demonstrate the applicability of Amoxi-TPP-AuNPs as a drug delivery system to improve therapeutic action.

## 1. Introduction

Recently, bacterial resistance to antibiotics, especially to commonly used types such as β-lactams, has become a global problem in the medical field, resulting in the failure of treatments and high death rates. The inefficiency of antibiotics, especially penicillin group antibiotics including amoxicillin, which has been a first line of treatment since the nineties due to its safety, has led to the rapid spread of bacterial resistance in pathogenic bacteria, including methicillin-resistant *Staphylococcus aureus* (MRSA), *Streptococcus pneumoniae*, *Klebsiella pneumoniae*, *E. coli*, and *Pseudomonas aeruginosa* [1,2,3,4]. An alarming report from the Centers for Disease Control and Prevention made the first mention of these bacteria, assigning them as an urgent danger or a critical concern regarding multi-drug resistance. The report called for prompt and forceful action to ensure that the situation does not worsen [5]. Amoxicillin is a β-lactam and penicillin antibiotic that prevents bacteria from building cell walls, leading to bacterial cell rupture and the release of their component parts, which results in the death of the bacteria. The fact that it secretes β-lactamase enzymes that break down the wall hinders its attempts to prevent building of the wall, leading to continued reproduction and infection. Urgent action is required to address this problem [6,7,8].

There is a pressing need for novel techniques to create new antibiotics, increase their efficacy, and discover strategies to overcome bacterial resistance. One of the most promising methods in modern medicine is the use of metal nanoparticles to deliver antibiotics, as they can be used to create active nano-antibiotics that are highly effective at killing MDR bacteria. Organic, inorganic, and composite materials can be found among the nanoparticles used in nanodrug delivery devices. The creation of drug-loaded nanomaterials is a popular biosynthesis strategy because it reduces the amount of potentially harmful components in the biosynthesis process. Additionally, using green nanoparticles to transport medicines can reduce their adverse effects [9]. A family of nanoparticles known as “nano-drug delivery systems” has the potential to enhance the stability and water solubility of drugs, extend their cycle time, speed up the absorption of drugs into target cells or tissues, and decrease enzyme degradation [10,11].

However, using these nanoparticles has some drawbacks, particularly when they are prepared with AuNPs without linkers [12], which decreases their effectiveness [13,14,15]. Physical, chemical, enzymatic, and biological processes can all be used to create metallic nanoparticles [16,17]. The physical and chemical processes used in their manufacture are potentially harmful to humans and the environment. Therefore, biological preparation techniques, particularly those that use plants, bacteria, fungi, and algae, are the best choice. One of the best sources of metallic nanoparticles is plants, such as *J. excelsa*, that have reducing compounds like terpenoids, flavonoids, phenols, and alkaloids; these compounds give these nanoparticles their durability [13,18]. The antibiotic amoxicillin is a β-lactam antibiotic and is used to treat bacterial infections, especially respiratory and urinary tract infections. It has effectiveness against a wide range of pathogenic bacteria and can inhibit bacterial wall synthesis. It is frequently used in medicine because it is more readily absorbed than other antibiotics [13].

For medicinal applications and human health, the biological properties of AuNPs are particularly relevant. The use of AuNPs has several important advantages, including high compatibility and safety, as well as their function as drug delivery agents [13]. Furthermore, drug encapsulation, surface covalent binding, and other non-covalent nano-assemblies make AuNPs an efficient loadable material [19]. Antibiotics are transported throughout the body with the help of molecules called drug carriers. Due to their “nano” size, AuNPs are suitable drug carriers due to their easy entry into various cells [19]. 

In the current study, Amoxi-TPP-AuNPs were prepared and used against MDR bacteria as an active nanodrug for treating various bacterial diseases. Amoxicillin was conjugated with safe and biologically prepared AuNPs that were synthesized from *Juniperus excelsa* (*J. excelsa*), which contains reducing and stabilizing agents. Sodium tri-polyphosphate (TPP) was used to conjugate amoxicillin to AuNPs, improving the efficiency and stability of the binding processes and the antibiotic’s mode of action. Amoxi-TPP-AuNP conjugate is a hybrid agent for the inhibition of MRSA strains and MDR *E. coli* strains, as they are β-lactamase-producing bacterial strains. Amoxi-TPP-AuNPs have a very low minimum inhibitory concentration (MIC), which adds novelty and significance to this study.

## 2. Materials and Methods

### 2.1. Preparation of Juniperus excelsa Extract

*J. excelsa* leaves were thoroughly cleansed with Milli-Q water until no foreign matter was left. With 100 mL of deionized water and 25 g of finely chopped *J. excelsa* leaves, the mixture was stirred at 85 °C for 20 min. Whatman No. 1 (Sigma Aldrich, St. Louis, MO, USA) filter paper was used to filter the *J. excelsa* leaf extract, and the filtrate was stored at 4 °C for use as a reducing agent and stabilizer in later experiments [20].

### 2.2. Biosynthesis of AuNPs

The *J. excelsa* leaf extract was added drop-by-drop to 50 mL of a 1 mM aqueous solution of HAuCl_4_3H_2_O at various temperatures and pH levels while being stirred continuously, according to the protocol of Gad El-Rab et al. [21]. There was a change in color related to AuNP formation. After prolonged reduction, the AuNPs were recovered by centrifuging at 15,000× *g* for 30 min. Then, de-ionized water was used to completely purify them, followed by 50% EtOH. Finally, the AuNP sample was held at ambient temperature (25 °C) until further analysis.

### 2.3. Factors Affecting the Synthesis of AuNPs

The following criteria were investigated based on research by Gad El-Rab et al. [18]:

#### 2.3.1. Effect of HAuCl_4_3H_2_O

UV-Vis spectroscopy was used to measure the effect of HAuCl_4_3H_2_O (Sigma, USA) at varied concentrations (1–10 mM). 

#### 2.3.2. Effect of *J. excelsa* Leaf Extract

Various quantities of the *J. excelsa* leaf extract (2, 4, 6, 8, and 10 mL) were used to assess how it affected the production of the AuNPs. 

#### 2.3.3. Effects of pH Levels

Additionally, different pH levels were applied to examine the pH-dependent effect (5.0, 6.0, 7.0, 8.0, 9.0, 10.0, and 11.0). 

#### 2.3.4. Effects of Temperatures

Different temperatures (25, 35, 45, 60, 80, and 100 °C) were applied to examine the effect of temperature on the biosynthesis of the AuNPs. 

#### 2.3.5. Effect of Incubation Time

The impact of the incubation period was assessed using a range of incubation intervals (0, 30, 60, 80, and 240 min).

### 2.4. Conjugation of Amoxicillin with AuNPs

Herein, a two-stage synthetic procedure was used to conjugate AuNPs (15.99–24.71 nm or 50.34–100.45 nm) to amoxicillin (Oxoid Ltd., Basingstoke, UK) to produce Amoxi-TPP-AuNPs. In a previous method, the initial step of the AuNP synthesis was undertaken by mixing 8 mL of *J. excelsa* leaf extract with 10 mL of 5 mM HAuCl_4_3H_2_O at pH 6 and 25 °C for 1 h. The second stage involved combining TPP (Sigma Aldrich, St. Louis, MO, USA) (1 mg mL^−1^ in PBS, pH = 6) with AuNPs (83 µg mL^−1^ in PBS, pH = 6) and shaking the mixture for 30 min to create TPP-AuNPs. Amoxicillin (1 mg mL^−1^) was dissolved in 1 mL of organic solvent dimethyl sulfoxide (DMSO), and then diluted to 5 mL with PBS (pH 6). The mixture was then agitated for 30 min at a 4 °C. To facilitate the binding process of the amoxicillin to the TPP-AuNPs, 1.5 mL of the previously manufactured amoxicillin was combined with 3.5 mL of the previously synthesized TPP-AuNPs, and the mixture was agitated in an ice bath (0 °C) for 1 h. The Amoxi-TPP-AuNPs were collected by ultracentrifugation at 12,000× *g* for 10 min. To eliminate any unreacted compounds, freshly synthesized Amoxi-TPP-AuNPs were dialyzed overnight against double-distilled water using a 10 kDa cellulose membrane (Sigma Aldrich, St. Louis, MO, USA). The samples were dialyzed and then freeze-dried. After being obtained, the Amoxi-TPP-AuNPs were kept at 4 °C for further characterization.

### 2.5. Characterization of AuNPs and Amoxi-AuNPs

The characterization of AuNPs and Amoxi-AuNPs was performed according to the method described by Gad El-Rab et al. [21].

#### 2.5.1. UV–Vis Spectroscopy

The UV-Vis spectra of the AuNPs and Amoxi-TPP-AuNPs were analyzed using a Shimadzu uv-1650 PC spectrophotometer (Osaka, Japan). By analyzing the UV-Vis spectra of the AuNPs and Amoxi-TPP-AuNPs in the wavelength range of 300–800 nm, the AuNPs and Amoxi-TPP-AuNPs were monitored. 

#### 2.5.2. TEM Analysis

The morphology and size of Amoxi-TPP-AuNPs and AuNPs were shown using TEM analysis. A JEOL 100CX II model electron microscope at Assiut University was used with an acceleration voltage of 100 kV to produce the TEM image [22,23,24]. The sample was prepared by pipetting aqueous Amoxi-TPP-AuNP or AuNP sample droplets onto carbon-coated copper grids. The film on the grid was then given time to dry. *E. coli* cells that had been treated with amoxi-TPP-AuNPs (6 µg/mL) were also imaged. After incubation, the bacterial suspension was centrifuged at 3000× *g* for 10 min. The pellets were acquired and then washed with PBS four times, each time for 30 min. After that, they were dyed for 3 h in the dark and at room temperature using osmic acid (3×). Next, the samples were fixed in 2.0% agar, and further samples from the agar were dehydrated using gradient doses of ethanol (70 for 30 min, 90 for 30 min, and 100% for 30 min), followed by 100% ethanol (Sigma Aldrich, St. Louis, MO, USA) overnight, and finally, by acetone (Sigma Aldrich, St. Louis, MO, USA) for 30 min. Following the embedding technique in a mold, the resultant solidified media was cut into thin tissue slices for microscopic analysis. For 45 min, another 45 min, and 1 h, each sample was immersed in a 0.5 mL gradient embedding solution (1:1 *v*/*v* ethanol: acetone; 2:1 and 100% ethanol). To finish, samples were put into a mold (0.3–0.5 mL) and heated to 70 °C for 48 h. Slices were made using an ultra-sectioning tool and then placed on gold grids for TEM analysis.

#### 2.5.3. X-ray Diffraction (XRD) Analysis

The AuNP crystal structure was interpreted using XRD (Shimadzu XD-3A, Tokyo, Japan).

#### 2.5.4. FT-IR Analysis

The solution of Amoxi-TPP-AuNPs and AuNPs was centrifuged at 12,000× *g* for 15 min. The unattached biological components from the surface of the nanoparticles were removed by washing the residue from earlier products with bi-distilled water. A spectrometer (Shimadzu IR-470, Tokyo, Japan) was used to quantify the FT-IR in the 4000–400 cm^−1^ region using the powder of the extract of *J. excelsa* leaves, AuNPs, TPP-AuNPs, and Amoxi-TPP-AuNPs [24]. In order to create discs, 100 mg of grade KBr was mixed with 100 mg of hydraulic pressure after the sample had been desiccated at 100 mg. The peak heights were represented by the FT-IR peak wavenumbers (cm^−1^) that were found.

### 2.6. Determination of Conjugating Efficiency of Amoxi-TPP-AuNPs

The conjugating efficiency of Amoxi-TPP-AuNPs was calculated according to the method described by Shaker and Shaaban [25]. The Amoxi-TPP-AuNPs underwent 12,000× *g* centrifugation for 15 min to remove free amoxicillin and TPP, and the residue was suspended in distilled water. A Shimadzu uv-1650 PC spectrophotometer (Osaka, Japan) was used to measure the amount of free amoxicillin in the supernatant, and an analysis was conducted according to the protocol of Güliz [26]. The linear range of the amoxicillin calibration curve was chosen to be 5–110 mg mL^−1^. Moreover, the peak of amoxicillin was obtained at λ max 341. We then had to find how much amoxicillin was linked to the AuNPs to determine the percentage of Amoxi-TPP-AuNP conjugating efficiency. The conjugating efficiency of Amoxi-TPP-AuNPs (%) was determined according to the following calculation:

Conjugating efficiency of Amoxi-TPP-AuNPs (%) = (initial amount of amoxicillin − free amoxicillin in the supernatant/initial amount of amoxicillin) × 100

### 2.7. In Vitro Drug Release Kinetics

The administration of amoxicillin involved membrane dialysis. Three millimeters of Amoxi-TPP-AuNPs (2 mg mL^−1^, in PBS, pH = 7.4) (15.99–24.71 nm or 50.34–100.45 nm) were added to a dialysis bag (MW = 12 KDa). The membrane bag was stored overnight at 37 °C in 30 mL of PBS saline (pH = 6.0 and 7.4) in a shaking water bath running at 100 rpm. To prevent the amoxicillin from deteriorating, the antioxidant ascorbic acid was added to the Amoxi-TPP-AuNPs solution (0.2% *w*/*v*). Additionally, amoxicillin also served as a control using the membrane dialysis technique. At predetermined times, 1 mL of the sample was taken out and replaced with fresh PBS medium. After measuring the sample’s absorbance at 341 nm with a UV-Vis spectrometer, calibration curves were plotted.

### 2.8. Isolation and Characterization of Clinical Isolates

#### 2.8.1. Collection of Clinical Isolates and Growth Conditions

The samples were obtained from individuals with wound infections at Al-Edwani Hospital in Taif, Saudi Arabia. Within 1 h of collection, the infected swabs were delivered to the lab for bacteriological investigation. The swabs were acquired by removing any contamination from the patient’s infected region with sterile cotton swabs. The infected swabs were put on the surface of several media, including blood agar (Oxoid Ltd., Basingstoke, UK), mannitol salt agar (Oxoid Ltd., Basingstoke, UK), and MacConkey agar (Oxoid Ltd., Basingstoke, UK) and incubated for 16 to 24 h at 37 °C. Gram-positive bacteria were discovered on sheep blood agar plates using a variety of analytical techniques, including catalysis reactions, Gram staining, hemolytic activity testing, and coagulase testing. Gram-negative bacteria were found using blood agar and MacConkey agar and biochemical processes based on the shape of their colonies on these media. Gram-negative bacteria were detected using biochemical techniques such as urease tests, citrate oxidase, triple sugar iron (TSI), sulfur, indole, and motility (SIM), and based on the appearance of their colonies on blood agar and MacConkey agar media [27]. The criteria of MaccFadin [28], Loberto et al. [29], Chaudhary et al. [30], morphological, biochemical, and API 20E Strep and API Staph test kits (BioMerieux, Craponne, France) were employed for *E. coli* and *S. aureus*. Single bacterial colonies were also discovered.

The most prevalent bacterial isolates were found to be methicillin-susceptible *S. aureus* (MSSR), *E. coli*, and methicillin-resistant *S. aureus* (MRSA). These bacterial strains were routinely preserved in the microbiology laboratory on Muller-Hinton agar slants at 4 °C. To study the antimicrobial effectiveness of AuNPs alone or in conjunction with antibiotics, cell suspensions were diluted with a sterile saline solution to a final concentration of 10^7^ CFU/mL. The tested strains were inoculated and grown in Mueller-Hinton broth with AuNPs or Amoxi-TPP-AuNPs for 16 to 24 h at 37 °C.

#### 2.8.2. Molecular Typing

##### Molecular Characterization Using the 16S rRNA Gene

The Wizard Genomic DNA Purification Kit (Promega, Madison, WI, USA) was used to recover genomic DNA from bacterial cells. Five milliliters of an overnight culture was mixed with 900 µL of 50 mM ethylene diamine tetra acetic acid (EDTA), and the mixture was centrifuged at 15,000× *g* for 3 min at 25 °C to resuspend the pellets. A volume of 120 µL of lysozyme solution was combined with the cell suspension (about 40 mg mL^−1^ of lysozyme solution, Sigma Aldrich, St. Louis, MO, USA), and the mixture was then incubated for 1 h at 37 °C in a water bath to break down the cell wall. The bacterial suspension was centrifuged at 15,000× *g* for 3 min at 25 °C to create a pellet. The nuclei lysis solution (Promega, Madison, WI, USA) was then added to 900 µL of the pellet and gently resuspended. After incubating the bacterial isolate cells at 80 °C for 5 min, the cells were lysed. Four microliters of RNase A (50 mg mL^−1^) (Sigma Aldrich, St. Louis, MO, USA) was then added to the RNA lysis. The tubes were inverted 10 times before the mixture was maintained again for 1 h at 37 °C. For the protein precipitation stage, 300 µL of precipitation solution (Promega, Madison, WI, USA) was added to the lysate mixture and vortexed for 30 s. The mixture was then chilled for 7 min before being centrifuged for 15 min at 15,000× *g*. The supernatant was transferred to an Eppendorf tube containing 600 µL of isopropanol at 25 °C for the washing stage. The mixture was then gently mixed by inverting the tube and then centrifuged at 15,000× *g* for 10 min. Additionally, 70% EtOH was used to clean the pellet. The genomic DNA pellet was resuspended in 60 µL of DNA rehydration solution as the last step (Promega, Madison, WI, USA).

##### PCR of 16S rRNA Genes

The template DNA (1 µL) was added to 20 µL of the polymerase chain reaction (PCR) solution. Then, 27 F (AGA GTT TGA TCM TGG CTC AG) and 1492 R (TAC GGY TAC CTT GTT ACG ACT T) were used as forward and reverse primers, respectively. Next, 35 cycles of 16S rRNA gene amplification were done at 94 °C for 45 s during the denaturation stage, at 55 °C for 60 s during the annealing step, and finally, at 72 °C for 60 s during the extension step. A 1400-bp DNA fragment was amplified.

##### 16S rRNA Gene Analysis

Sequencing was done on the approximately 1400 bp-sized purified 16S rRNA PCR products using forward primer 518F (5′-CCA GCA GCC GCG GTA ATA CG-3′) and reverse primer 800R (5′-TAC CAG GGT ATC TAA TCC-3′). A Big Dye Terminator Cycle Sequencing Kit (Applied Biosystems, Foster City, CA, USA) was used to finish the sequencing. An automated DNA sequencing tool from Applied Biosystems, model 3730XL, was used to analyze the sequencing results. After performing a sequencing analysis, the CLUSTAL W (1.81) phylogenetic tree was used to assess similarities among the bacterial strains. The 16S rRNA gene sequences of the isolates described herein were uploaded to the nucleotide sequence databases DDBJ/EMBL/GenBank.

### 2.9. Antibiotic Susceptibility Testing

#### Disk Diffusion Method

The isolates were grown in peptone water at 37 °C. Bacterial cultures (100 μL 1.0 × 10^4^ CFU/mL) were spread on the surface of Mueller-Hinton agar plates. Different amounts of antibiotic were dissolved in 30 μL water and then dropped onto the filter discs. Antibiotic discs were positioned on Mueller Hinton agar plates, where the bacterial culture was being grown. At 37 °C, the plates were incubated for 18 h. The inhibition zones were described using the recommendations of the Clinical Laboratory Standard Institute (CLSI) [31]. Thirty-one different antibiotics (Oxoid Ltd., Basingstoke, UK) were used to assess the susceptibility of bacterial isolates. These antibiotic were Amikacin (AK), amoxicillin-clavulanic acid (AMC), Cefaclor (CEC), Cefotaxime (CTX), Cefoxitin (FOX), Ceftazidime (CAZ), Cefuroxime (CXM), Doxycycline/HCL (DO), Ciprofloxacin (CIP), Levofloxacin (LVX), Clarthromycin (CLR), Ampicillin (AMP), Cefalexin (LEX), Cefadroxil (FEP), Cefaclor (CEC), Ceftriaxone (CRO), Meropenem (MEM), Sulpha/Trimethoprim (SXT), Tigecycline (TGC), Gentamicin (GEM), Imipenem (IPM), Nitrofurantoin (NTF), Fluxocillin (FUX), Azithromycin (AZM), Clindamycin (CLI), Penicillin (P), Oxacillin (OXA), Erythromycin (ERY), Vancomycin (VAN), and Rifampicin (RIF).

### 2.10. Antibacterial Activity of Amoxi-TPP-AuNPs Using the Well Agar Diffusion Method

The antimicrobial efficacy of solutions containing AuNPs (83 µg mL^−1^), TPP-AuNPs (1 mg mL^−1^: 83 µg mL^−1^), amoxicillin (30 µg mL^−1^), and Amoxi-TPP-AuNPs (6 µg mL^−1^) was tested using the well agar diffusion method [25]. Bacterial cultures (100 μL 1.0 × 10^4^ CFU/mL) were initially injected into nutrient broth medium and then incubated for 18 h at 37 °C. Consequently, 6 mm wells were made on nutrient agar plates and Mueller-Hinton agar media after inoculation with 100 µL of each bacterial culture. Different concentrations of AuNPs (83 µg mL^−1^), TPP-AuNPs (1 mg mL^−1^: 83 µg mL^−1^), amoxicillin (30 µg mL^−1^), and Amoxi-TPP-AuNPs (6 µg mL^−1^) were pipetted into the wells. After that, the plates were incubated for 24 h at 37 °C. The inhibition zone was recorded by measuring the diameter of the clear area surrounding each well.

### 2.11. Determination of Minimum Inhibitory Concentration (MIC) and Minimum Bactericidal Concentration (MBC) of Amoxi-TPP-AuNPs

The minimum inhibitory concentration (MIC) and minimum bactericidal concentration (MBC) assessments were performed on a 96-well plate. Each well in column 1 received 200 µL of amoxicillin or Amoxi-TPP-AuNPs (15.99–24.71 nm or 50.34–100.45 nm). One hundred microliters of broth medium were injected into the wells in each row. Following that, 100 µL of either the amoxicillin (57–228 µg mL^−1^) or amoxi-TPP-AuNPs (2–64 µg mL^−1^) solution was drawn from column 1 and serially diluted along the row to column number 10. Column number 10 was regarded as a blank. Five microliters of the bacterial cultures were then pumped into each well containing the appropriate media. Additionally, each well received 5 µL of phenol red dye (2 mg mL^−1^) to gauge the vitality of the bacteria. A 96-well plate was incubated, and after 24 h at 37 °C, the presence of viable red bacterial cells was found. The growth was identified by the absence of color in the well. The MIC values of Amoxi-TPP-AuNPs or amoxicillin corresponded to the lowest concentrations at which no growth or color was seen. On the surface of agar medium plates, 5 µL from each well were inoculated, and the MIC and MBC was then measured. Bacterial culture plates were incubated for 24 h at 37 °C. The agar medium plates were then checked to see if there had been any growth. The MBC was assessed as having no growth [25].

### 2.12. Amoxi-TPP-AuNP Time Kill Test

Time-kill assays of amoxicillin and Amoxi-TPP-AuNPs were performed on *S. aureus* (MRSA) and *E. coli* and compared to the control, i.e., untreated organisms, in order to ascertain the rate of killing amoxicillin-resistant bacteria. Initially, the strains were grown in Mueller-Hinton broth medium at a concentration of 5 × 10^6^ CFU/mL. Following this, to the bacterial suspension was added either amoxicillin (30 g/mL) or Amoxi-TPP-AuNPs (8 g/mL) for *S. aureus* (MRSA) and for *E. coli*; the mixtures were then incubated at 37 °C. To count the remaining living bacteria using the surface drop method, samples were taken at intervals of 0, 1, 2, 3, 4, 6, 8, 10, and 12 h and diluted at a 1:10 ratio [32]. Using Mueller-Hinton agar medium, the numbers of bacteria belonging to *S. aureus* (MRSA) and *E. coli* were estimated. The mean numbers of viable bacteria were counted and displayed against the incubation period following the completion of three separate experiments in triplicate.

## 3. Results

### 3.1. Biosynthesis of AuNPs

**Figure 1:**
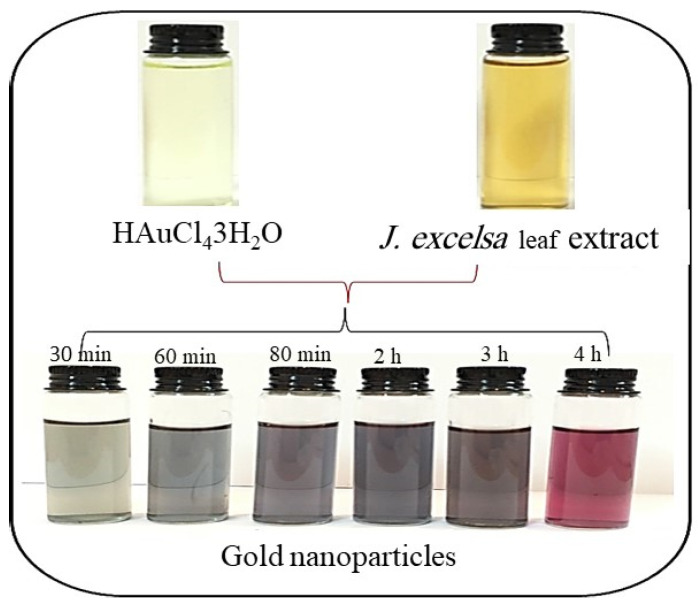
Change in color of the solution with time when HauCl_4_3H_2_O was added to *J. excelsa* leaf extract.

Within 30 min of adding the HAuCl_4_3H_2_O solution to the *J. excelsa* extract, the color changed from colorless to wine red, suggesting the synthesis of AuNPs (Figure 1). This is because the *J. excelsa* extract has biological reducing agents that aid in the production of AuNPs. The AuNP solution turned red as a result of surface plasmon resonance (SPR) vibrations. It was found that the entire color change took around 1 h; the color of the reaction mixture remained unchanged after that. This demonstrated a decrease in the concentration of HAuCl_4_3H_2_O in the reaction mixture.

#### 3.1.1. Effect of *J. excelsa* Extract Volume on AuNP Formation

**Figure 2:**
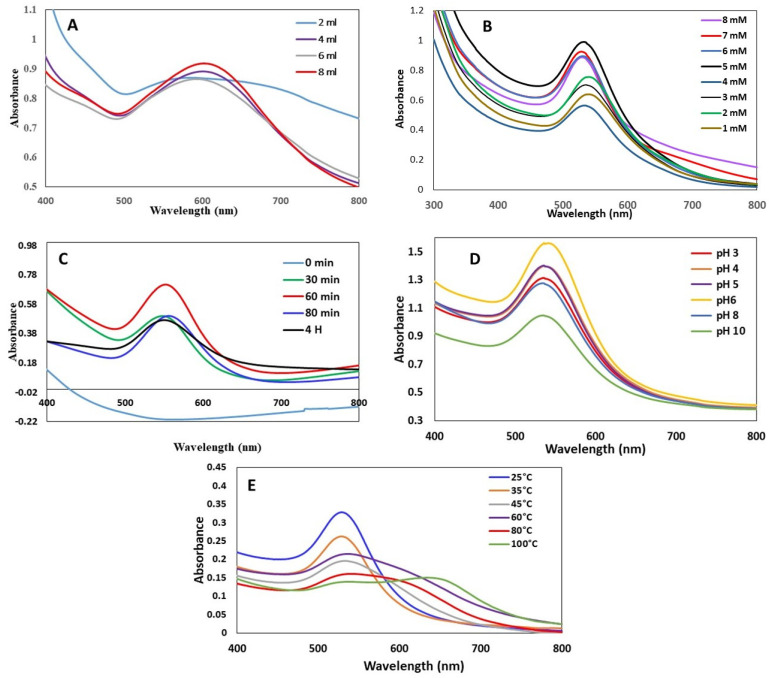
Effect on the formation of AuNPs using of *J. excelsa* leaf extract as recorded by the UV-Vis spectroscopy with (**A**) *J. excelsa* leaf extract volume at pH 6, 10 mL of 2 mM HAuCl_4_3H_2_O, 1 h, and 25 °C; (**B**) HAuCl_4_3H_2_O concentration at pH 6, 8:10 ratio of extract, 1 h, and 25 °C; (**C**) time at pH 6, 8 mL extract, 10 mL of 5 mM HAuCl_4_3H_2_O, and 25 °C; (**D**) pH at 8 mL of extract, 10 mL of 5 mM HAuCl_4_3H_2_O, 1 h, and 25 °C; (**E**) temperature at 8 mL of extract, 10 mL of 5 mM HAuCl_4_3H_2_O, 1 h, and pH 6.

Figure 2A shows the increase in AuNP absorption spectra by increasing the amounts of *J. excelsa* extract with HAuCl_4_3H_2_O (10 mM). The observed broadening of peaks was due to the higher concentration of HAuCl_4_3H_2_O (10 mM). A substantial absorption peak was found when the volume ratio of HAuCl_4_3H_2_O to *J. excelsa* leaf extract was 10:2. When the reaction mixture had a 10:8 ratio of HAuCl_4_3H_2_O to leaf extract, the highest intensity rapidly rose, indicating an increase in the creation of AuNPs. The intensity of absorption increased monotonically as the volume of the *J. excelsa* extract increased (Figure 2A). Additionally, the absorbance spectrum narrowed when the *J. excelsa* leaf extract volume increased (10:8), showing an increase in nanoparticle concentration and a reduction in AuNP size.

#### 3.1.2. Effect of HAuCl_4_3H_2_O Concentration on AuNP Formation

The AuNP absorption spectra produced by reducing various HAuCl_4_3H_2_O concentrations with *J. excelsa* leaf extract are shown in Figure 2B. The HAuCl_4_3H_2_O concentrations used for AuNP production were between 1 and 8 mM. The strength of absorption increased as HAuCl_4_3H_2_O concentration increased. This altered the maximum absorption position, indicating that the particle size was influenced by the HAuCl_4_3H_2_O concentration. When the HAuCl_4_3H_2_O concentration was increased from 1 to 5 mM, the absorption became more intense. As the HAuCl_4_3H_2_O concentration was increased above this concentration, the strength of the absorption decreased. As the precursor concentration was elevated from 1 to 5 mM, there was a small blue shift in the SPR wavelength, from 535 to 526 nm, which suggested that the mean particle diameter of the produced AuNPs had decreased while the mean particle diameter of the produced AuNPs had increased as the HAuCl_4_3H_2_O concentration was increased from 5 mM to 9 mM. Furthermore, the absorbance peak of AuNPs decreased as the HAuCl_4_3H_2_O concentration was increased by 5 mM, indicating particle precipitation, aggregation, and instability.

#### 3.1.3. Effect of Time on AuNP Formation

Figure 2C depicts the AuNP absorption spectra, which were derived from the reaction of *J. excelsa* leaf extract with HAuCl_4_3H_2_O at various reaction times (0–240 min). The absorbance increased with the passage of time (Figure 2C), showing an increase in the synthesis of AuNPs. Initially, a color change was detected after 10 min of applying the HAuCl_4_3H_2_O solution to the *J. excelsa* leaf extract solution. After 60 min, the hue of the solution became constant, suggesting that no HAuCl_4_3H_2_O remained for further reaction. After 60 min, the absorption peak decreased with increasing time and a change in peak wavelength.

#### 3.1.4. Effect of pH on the AuNP Formation

The remedy pH of the reaction played a significant role in the creation of AuNPs. As the pH fluctuated, the particle shape and size altered as well. The capping and stabilizing abilities of biomolecules may be impacted by pH, since this can alter their charge. Variations in peak wavelength and intensity were observed when the solution pH changed (Figure 2D). pH 6 increased the absorption maximum to 534 nm. This proved that pH 6 was the ideal pH for AuNP synthesis using a leaf extract of *J. excelsa*. pH 8 inhibited AuNP production (Figure 2D).

#### 3.1.5. Effect of Temperature on the AuNP Formation

Temperature was found to be an essential factor in the creation of AuNPs. Figure 2E depicts the AuNP absorption spectra at a variety of temperatures ranging from 25 to 100 °C. The HAuCl_4_3H_2_O reduction was accelerated, as shown by a change in the color of the solution at 25 °C. The spectra intensity decreased as the temperature increased from 25 to 100 °C. This indicates that 25 °C was the best for AuNP production.

### 3.2. Bio-Fabrication of Amoxi-TPP-AuNPs

**Figure 3:**
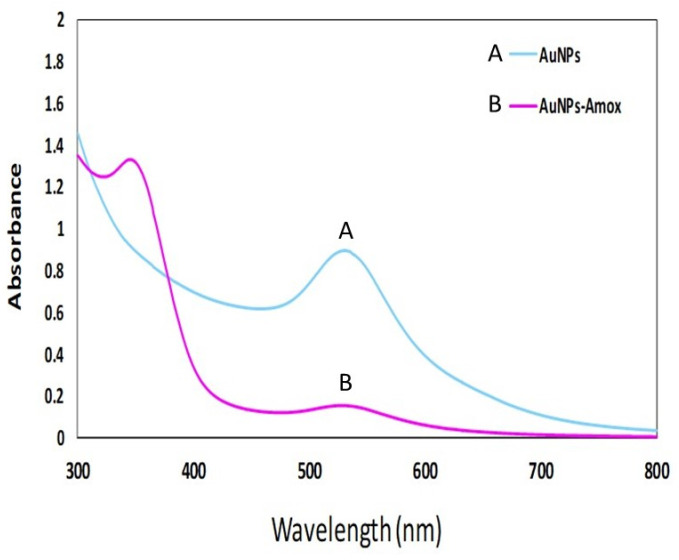
UV–Vis absorption spectra of biogenic AuNPs (A) synthesized using *Juniperus excelsa* leaf extract and Amoxi-TPP-AuNP conjugate (B).

The AuNPs were produced at optimum conditions of pH 6, 5 mM HAuCl_4_3H_2_O, and 25 °C for one hour. Moreover, the amoxicillin was conjugated with AuNPs using TPP. One peak was observed at 534 (Figure 3A) that related to AuNPs, while in the case of Amoxi-TPP-AuNPs (Figure 3B), two peaks appeared at 534 and 341 nm for AuNPs and amoxicillin, respectively. These two absorption peaks might have been caused by the positively charged amoxicillin amine group conjugating with the phosphate group of the TPP linker on the surface of the TPP-AuNPs.

**Figure 4:**
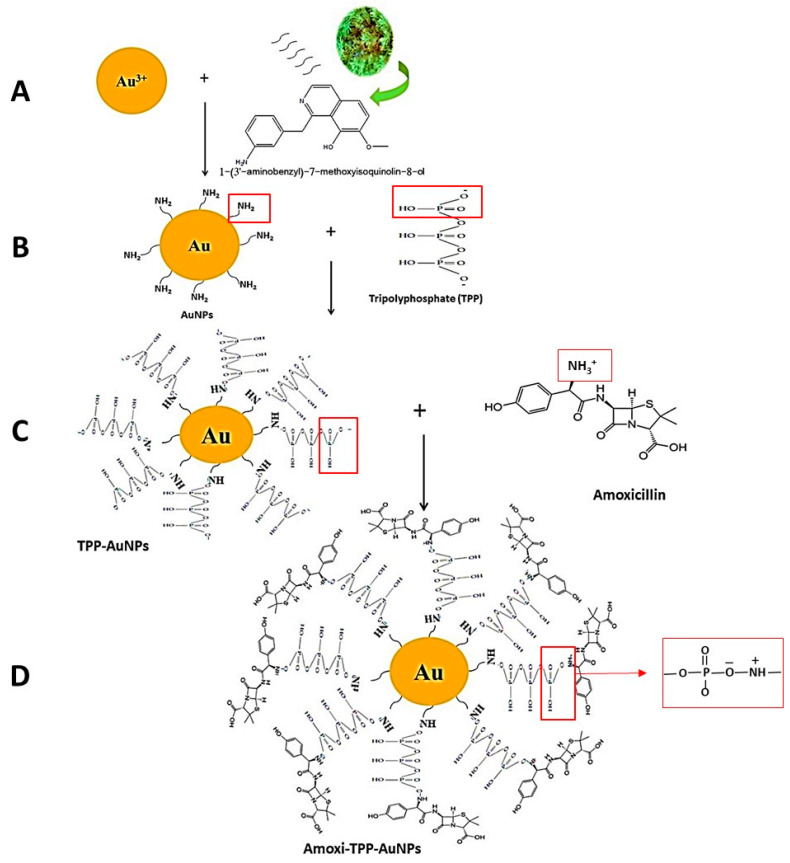
Schematic of the biosynthesis of Amoxi-TPP-AuNPs by conjugating amoxicillin with AuNPs using TPP as a linker.

Volatile oils, essential oils, alkaloids, glycosides, organic acids, oligosaccharides, resins, esters, phenolics, and alcohols are all included in the natural chemical makeup of *J. excelsa* leaf extract. A 4.6% extract of *J. excelsa* was found to contain aminoalcohol 3-1-benzylamine-7-methoxyisoquinoline-8-alcohol (1-(3′-aminobenzyl)-7-methoxyisoquinolin-8-ol) [29]. *J. excelsa* is involved in the reduction and creation of AuNPs (Figure 4A). Amoxi-TPP-AuNPs were found to be more stable and effective because the TPP linker improved the binding strength of amoxicillin with AuNPs by binding to both the amine groups of the AuNPs and the amoxicillin (Figure 4B–D).

### 3.3. AuNP and Amoxi-TPP-AuNP Characterization

#### 3.3.1. TEM with High Resolution

**Figure 5:**
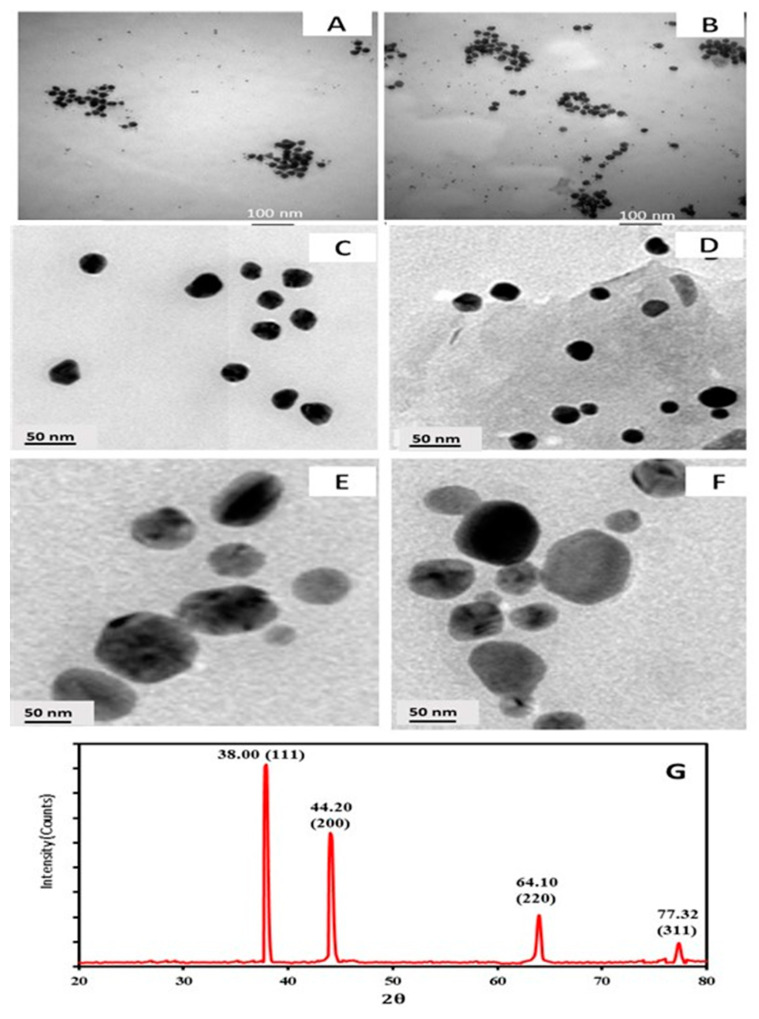
TEM images of AuNPs (15.99 to 24.71 nm) synthesized using *Juniperus excelsa* leaf extract and 5 mM HAuCl_4_3H_2_O (**A**,**C**), Amoxi-TPP-AuNP conjugate (15.99 to 24.71 nm) (**B**,**D**), AuNPs (50.34 to 100.45 nm) synthesized using *Juniperus excelsa* leaf extract and 9 mM HAuCl_4_3H_2_O (**E**), and Amoxi-TPP-AuNP conjugate (50.34 to 100.45 nm) (**F**), XRD analysis of synthesized AuNPs using *Juniperus excelsa* leaf extract (**G**).

TEM methods were used to detect the size and form of AuNPs and Amoxi-TPP-AuNPs. According to Figure 5A–F, AuNPs and Amoxi-TPP-AuNPs were successfully biosynthesized. It appeared that the AuNPs and Amoxi-TPP-AuNPs were round and hexagonal. The sizes of the synthesized AuNPs and Amoxi-TPP-AuNPs ranged from 15.99 to 24.71 nm under optimum conditions of pH 6, 8 mL extract, 25 °C, and 10 mL of 5 mM HAuCl_4_3H_2_O. The average size of the AuNPs was 20.35 nm, with a standard deviation (SD) of ±1.57 nm. Moreover, the particle size ranged from 50.34 to 100.45 nm under optimum conditions of pH 6, 8 mL extract, 25 °C, and 10 mL of 9 mM HAuCl_4_3H_2_O, with an average size of 75.40 ± 1.72.

#### 3.3.2. XRD Analysis

Figure 5G illustrates the matching X-ray diffraction (XRD) patterns used to examine the crystallinity of the synthesized AuNPs. Four different peaks at 2θ = 38, 44.2, 64.0, and 77.32 were seen in gold nanocrystals. All four peaks were consistent with typical Bragg reflections of the face center cubic (fcc) lattice, i.e., (111), (200), (220), and (311). The strong diffraction at peak 38.0 demonstrated that preferred growth orientation of the zero-valent gold was locked in the (111) direction. Pure Au nanocrystals often exhibit this XRD pattern.

#### 3.3.3. FTIR Analysis

**Figure 6:**
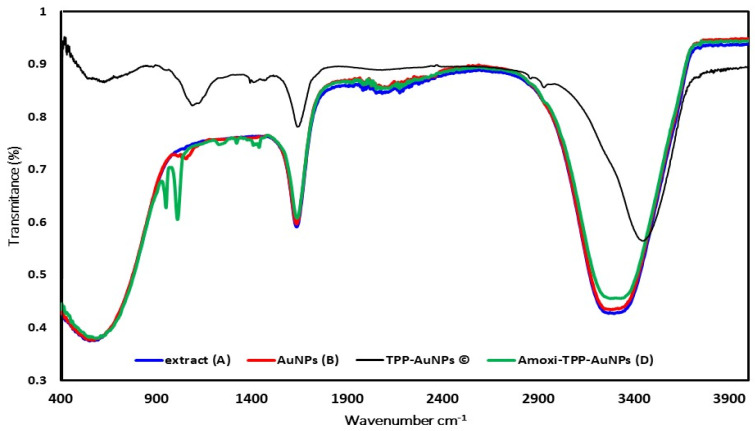
FTIR analysis of Extract (A), AuNPs (B), TPP-AuNPs (C) and Amoxi-TPP-AuNPs (D) from 4000 to 500 cm^−1^.

The FTIR spectrum of the extract revealed strong peaks at 3282, 1634, 1054, and 557 cm^−1^, which were associated with O-H stretching, N-H stretching, C-O-C stretching, and -C=C-H stretching, respectively (Figure 6A). Major peaks in the AuNP FTIR spectrum (Figure 6B) were seen at 3294, 1633, and 550 cm^−1^. These peaks corresponded to the O-H stretching peak, the N-H stretch, and 1-(3′-aminobenzyl)-7-methoxyisoquinolin-8-ol, an amino-alcohol component of *J. excelsa* leaf extract, respectively. The amine peak at 1633 cm^−1^ and the -C = C-H peak at 550 cm^−1^ were made stronger during the manufacture of AuNPs (Figure 6B). In the case of AuNPs, a peculiar peak at 3294 cm^−1^, denoting O-H stretching or H-bonding for alcohol or phenols, was observed.

FTIR spectra of TPP-AuNPs and Amoxi-TPP-AuNPs were obtained. The O-H stretching peak, the N-H stretch, the P-O group, and the -C=C-H, respectively, were revealed by TPP-AuNPs at 3449, 1636, 1093, and 624.5 cm^−1^. The O-H stretching peak was detected at 3449 cm^−1^. N-H group bending vibration was identified as the cause of the peak at 1636 cm^−1^. A strong peak at 1093 cm^−1^ was attributed to P-O stretches in TPP. When TPP was coated onto the AuNPs, the peak above was detected at 1010 cm^−1^ (Figure 6C). These findings clarify the existence of NH groups on AuNPs and their interactions with the anionic group of TPP. According to the peaks identified by FTIR analysis, TPP-AuNPs were formed when the P-O of TPP was joined to the NH groups of AuNPs. Two peaks at 1010.03 and 950.12 cm^−1^ in the FTIR of Amoxi-TPP-AuNPs were due to the cross-linking of amoxicillin amine groups with the P-O group of TPP-AuNP molecules (Figure 6D). These results suggest that a TPP linker facilitates the conjugating of AuNP and amoxicillin.

### 3.4. Determination of Amoxi-TPP-AuNP Conjugating Efficiency

The conjugating efficiency is a necessary parameter for the characterization of Amoxi-TPP-AuNPs at different nanoparticles sizes (15.99–24.7 nm and 50.34–100.45 nm). The efficiencies of Amoxi-TPP-AuNPs were determined to be 79.5% and 62.7%, respectively. The maximum concentration of amoxicillin and the highest conjugating efficiency were found in the smallest AuNP size sample, and as AuNP size increased, the conjugating percentage also decreased. Therefore, a strong conjugating effectiveness is essential to prevent amoxicillin from being lost during the creation of Amoxi-TPP-AuNPs; this formulation may lead us to use low doses of the drug for therapeutic purposes.

### 3.5. In Vitro Drug Release

**Figure 7:**
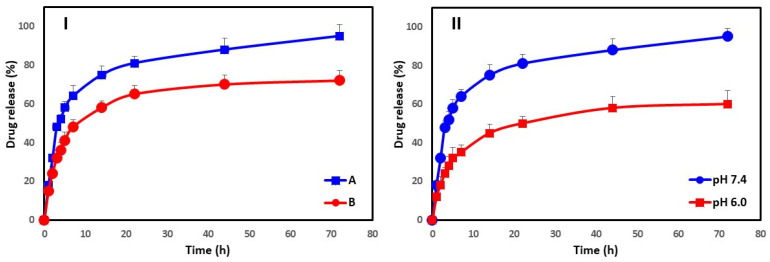
Drug release (%) of amoxicillin from Amoxi-TPP-AuNPs depends on size, i.e., (**I**) 15.99–24.71 nm (IA) and 50.34–100.45 nm (IB), and on pH (**II**) in PBS (pH = 6.0 and 7.4) at 37 °C.

Figure 7I,II displays the release of amoxicillin from Amoxi-TPP-AuNPs at different AuNP size (15.99–24.71 nm and 50.34–100.45 nm) and different pH. In Figure 7I, the Amoxi-TPP-AuNPs demonstrated a release profile that was characterized by an initial fast release rate (76% and 61%, respectively) which remained for the first 12 h, followed by a steady release phase with a slow-release rate, which lasted up to 3 d, ending in the complete release of the loaded amoxicillin. As a result, the amoxicillin release from Amoxi-TPP-AuNPs reached 83% and 75%, respectively, after 24 h and almost 90% and 81%, respectively, after 3 days. The AuNP size is responsible for this phenomenon. Since smaller AuNPs have a greater surface area to volume ratio, they contain more amoxicillin per unit volume. Furthermore, AuNPs with bigger sizes conjugated less amoxicillin for the same volume of particles. This results in less attraction between amoxicillin molecules and AuNPs. As a result, altering the size of AuNPs offers a method of regulating the release rates of amoxicillin. In Figure 7II, we see an increase in amoxicillin release with an increase in pH from 6 to 7.4.

### 3.6. Isolation and Identification of E. coli and S. aureus from Different Sources

The 78 isolates were collected from skin and urine. Urinary tract infections (UTIs) accounted for over 90% of all bacterial isolates, while skin infections accounted for 10% of all bacterial isolates (SKI). All isolates were identified based on their morphology and biochemistry (Table 1). *E. coli* is the prevalent and etiologically important bacterium found in urine, and it is also known to be the most common source of UTIs. *E. coli* was designated as *E. coli* ESC1, *E. coli* ESC2, *E. coli* ESC3, *E. coli* ESC4, *E. coli* ESC5, *E. coli* ESC6, *E. coli* ESC7, *E. coli* ESC8, *E. coli* ESC9, *E. coli* ESC10, and *E. coli* ESC11.

Nine *S. aureus* isolates were designated as STA1, STA2, STA3, STA4, STA5, STA6, STA7, STA8, and STA9 and were collected using sputum, high vaginal swabs (HVS), nasal swabs, and urine. Using information from each isolate’s appearance and biochemistry, *S. aureus* was identified (Table 1). About 55% of isolates were from respiratory tract infections (RTI), 22% were from UTIs, and 23% were from HVS (skin infections).

### 3.7. Antimicrobial Susceptibility by Disk Diffusion Method

The 11 bacterial isolates of *E. coli* recovered from urine and skin were subjected to antimicrobial agent sensitivity testing (Table 2). The collected results were interpreted in accordance with CLSI recommendations. Isolates ESC1, ESC2, ESC3, ESC4, ESC5, ESC6, ESC7, ESC8, ESC9, ESC10, and ESC11 were, respectively, resistant to 10, 10, 19, 9, 19, 8, 15, 10, 11, and 11 antimicrobial agents (Table 2). Eight different kinds of antimicrobial drugs (Ampicillin, Amoxycillin/Clavulanic Acid, Clarithromycin, Doxycycline/HCL, Cefalexin, Azithromycin, Ceftriaxone, and Fluxocillin) could not be used against all the *E. coli* isolates. Despite these findings, the isolates were susceptible to amikacin, piperacillin-tazobactum, nitrofurantoin, and imipenem; 75% of these isolates were MDR. Moreover, these isolates had high resistance rates to commonly used antimicrobial agents. Nine *S. aureus* isolates also recovered from urine, sputum, a high vaginal swab, and a nasal swab underwent antimicrobial agent sensitivity testing. Isolates STA1, STA2, STA3, STA4, STA5, STA6, STA7, STA8, and STA9 were, respectively, resistant to 5, 5, 15, 14, 11, 4, 9, 13, and 6 antibiotics (Table 2). Four different antibiotics (Cefaclor, Penicillin, Erythromycin, and Fluxocillin) could not be used to treat any infections of *S. aureus* strains (Table 2). Our findings showed that amikacin, fluxocillin, and trimethoprim-sulfamethoxazole were effective treatments for these bacterial isolates. The *E. coli* and *S. aureus* strains were multidrug resistant.

### 3.8. Molecular Characterization of E. coli and S. aureus Isolates from Different Sources 16S rRNA Analysis

**Figure 8:**
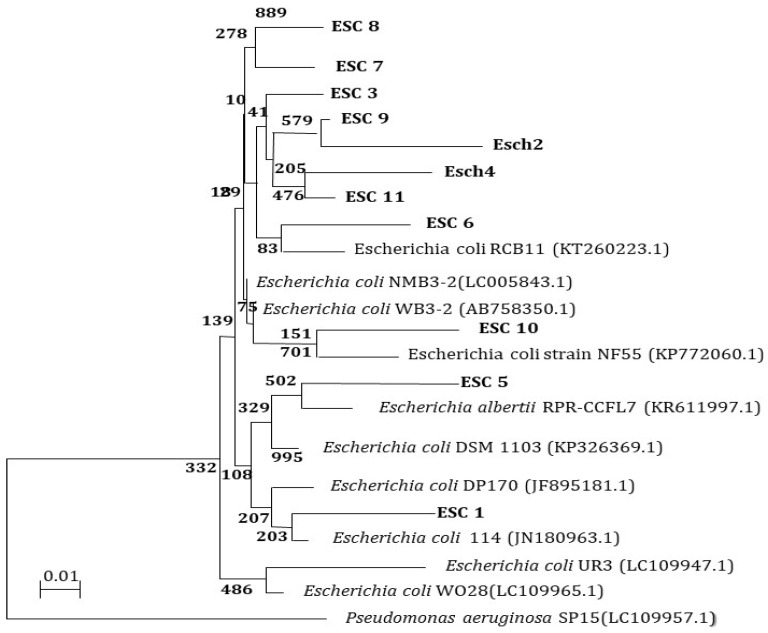
A phylogenetic tree of MDR *E. coli* isolates relied on the nucleotide sequences of 16S rRNA genes, constructed by the neighbor-joining method. The scale bar shows the genetic distance. The number presented next to each node shows the percentage bootstrap value of 1000 replicates.

The 16S rRNA encoding genes of 11 MDR *E. coli* and 9 MDR *S. aureus* isolates were PCR-amplified and sequenced for further characterization (Figure 8). The DDBJ/EMBL/GenBank nucleotide sequence databases received the 16S rRNA sequences of the isolates (ESC1, ESC2, ESC3, ESC4, ESC5, ESC6, ESC7, ESC8, ESC9, ESC10, and ESC11), which were assigned accession codes LC189092, LC189093, LC189094, LC189095, LC189096, LC189097, and LC189098, respectively. The nucleotide sequences of the MDR bacterial isolates were compared to sequences already in the databases. Figure 8 depicts a dendrogram that summarizes the findings of the 16S rRNA investigation. According to the results, the *E. coli* isolates had the best similarity to the other isolates. The 16S rRNA sequences of the isolates were shown to be most closely related to *E. coli*. This finding suggests that MDR bacterial isolates ESC1, ESC2, ESC6, ESC7, ESC9, ESC10, and ESC11 were heterogeneous, and thus, that these isolates were new strains.

**Figure 9:**
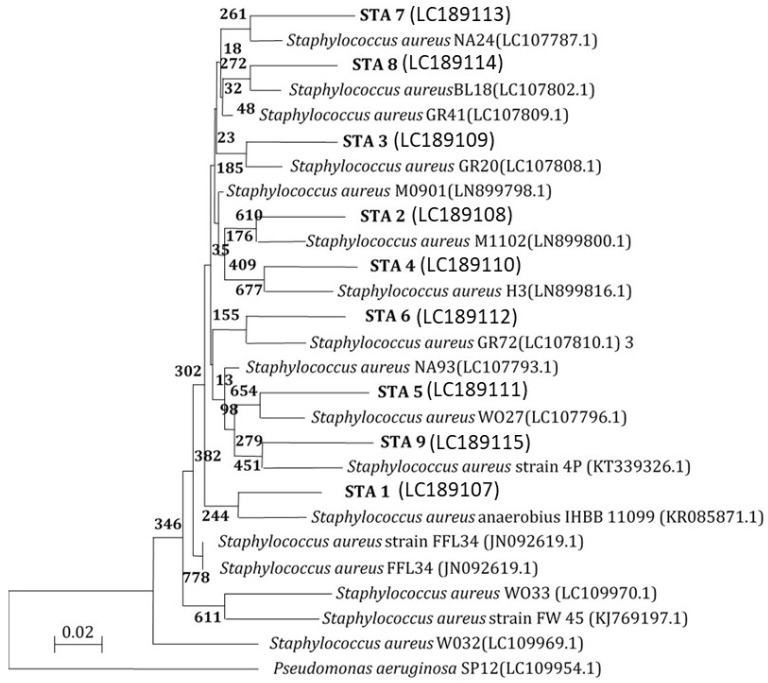
A phylogenetic tree of MDR *S. aureus* isolates, relying on the nucleotide sequences of 16S rRNA genes, was constructed by the neighbor-joining method. The scale bar shows the genetic distance. The number presented next to each node shows the percentage bootstrap value of 1000 replicates. The *Pseudomonas aeruginosa* SP12 was treated as the out-group. The GenBank accession numbers of the bacteria are presented in parentheses.

Next, 16S rRNA encoding genes of MDR bacterial isolates STA1, STA2, STA3, STA4, STA5, STA6, STA7, STA8, and STA9 were also PCR-amplified and sequenced. The 16S rRNA sequences of the isolates were deposited in the DDBJ/EMBL/GenBank nucleotide sequence databases with accession numbers LC189107, LC189108, LC189109, LC189110, LC189111, LC189112, LC189113, LC189114, and LC189115, respectively. The nucleotide sequences of the MDR bacterial isolates were compared to existing sequences in the databases. A dendrogram demonstrating the results of the 16S rRNA analysis is shown in Figure 9. According to the results, the isolated STA1, STA2, STA3, STA4, STA5, STA6, STA7, STA8, and STA9 had the strongest correlation to the *Staphylococcus* group. The 16S rRNA sequences of the *Staphylococcus* isolates were shown to be closely related to *S. aureus*. The isolates were diversified, as shown the phylogenetic tree. These data suggest that the bacterial isolates in this study were novel strains.

### 3.9. Inhibition Zone, Minimum Inhibitory Concentration (MIC), and Maximum Bactericidal Concentration (MBC) of AuNPs and Amoxi-TPP-AuNPs against MDR E. coli and S. aureus Strains

**Figure 10:**
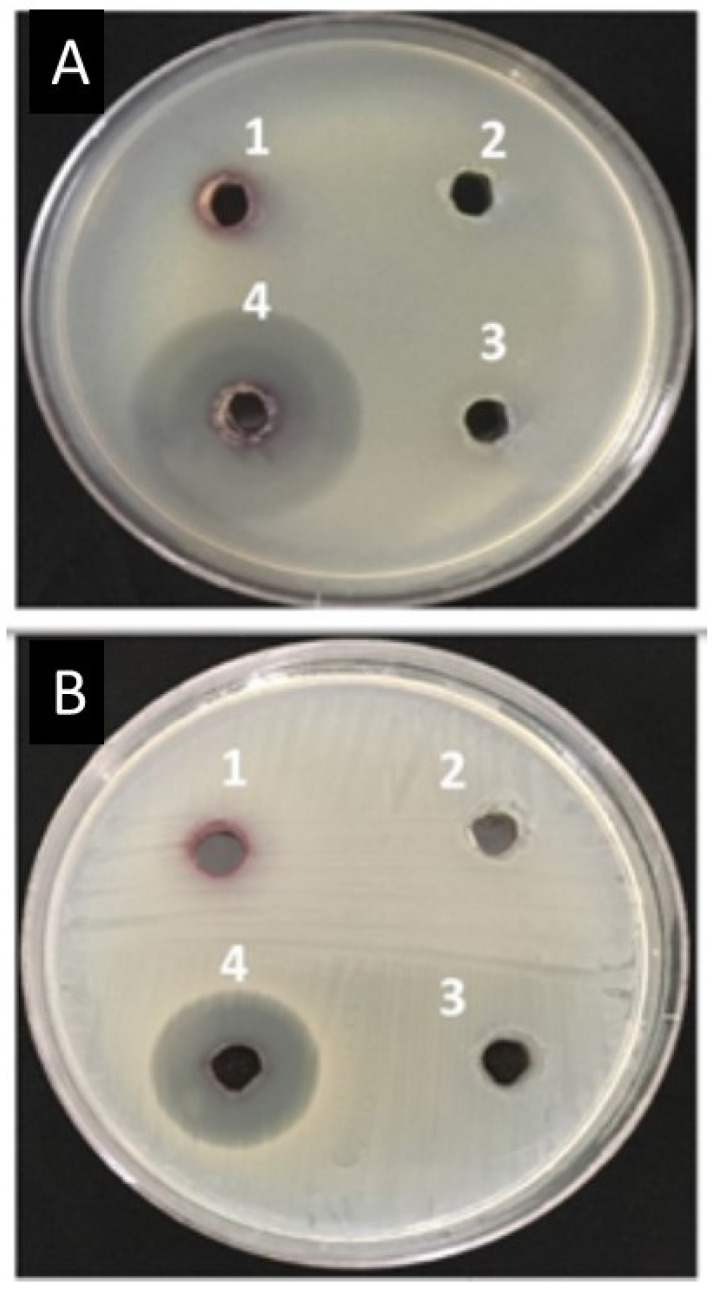
Antibacterial effects against *E. coli* (**A**) and *S. aureus* (**B**) isolates; inhibition zone of 1-AuNPs (83 µg/mL), 2-TPP-AuNPs (1 mg/mL: 83 µg/mL), 3-Amoxicillin (30 µg/mL), and 4-Amoxi-TPP-AuNPs (6 µg/mL).

Using well diffusion and micro-dilution plate techniques, biosynthesized AuNPs were tested for their antibacterial efficacy against pathogenic bacteria that were multidrug resistant. The synthesized AuNPs had no inhibition zone against bacteria that were resistant to several drugs, whereas the Amoxi-TPP-AuNP inhibition zone was 20–37 mm (Table 3 and Figure 10). Conversely, the TPP-AuNPs did not display an inhibitory zone, indicating that the antibacterial activity was a result of the slow release of loaded amoxicillin from the Amoxi-TPP-AuNPs.

The MIC of amoxicillin ranged from 96 to 114 µg mL^−1^ for the tested strains. The MIC of Amoxi-TPP-AuNPs (15.99–24.71 nm) against MDR strains was 3.6–8 µg, which was 12–31 times lower than the MIC of amoxicillin. The MBC of amoxicillin-TPP-AuNPs against MDR strains of *E. coli* and *S. aureus* was 12–32 µg mL^−1^.

Moreover, Amoxi-TPP-AuNPs conjugates and amoxicillin specifically acted as antimicrobials in the following order: Amoxi-TPPi-AuNPs (15.99–24.71 nm) > Amoxi-TPPi-AuNPs (50.34–100.45 nm) > amoxicillin, as shown in Table 4.

### 3.10. Mode of Action

**Figure 11:**
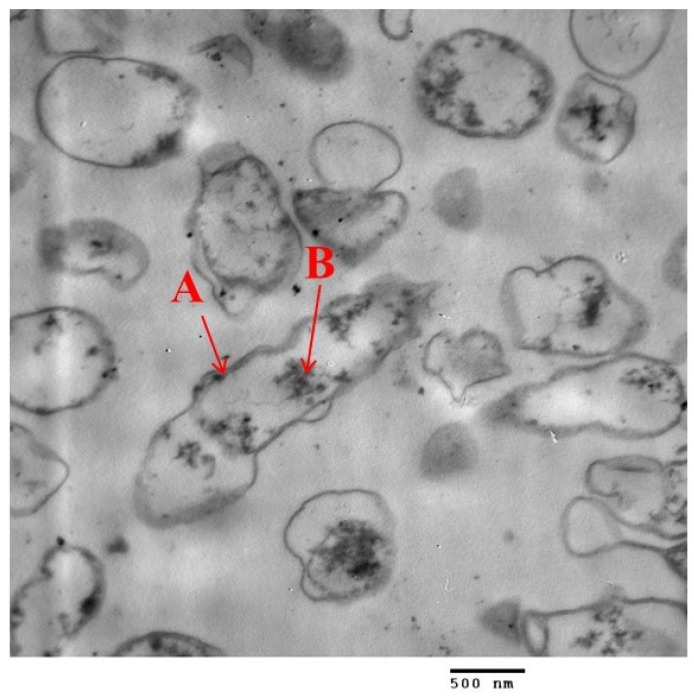
A TEM of *E. coli* ultra-section after exposure to (6 µg/mL) of Amoxi-TPP-AuNPs illustrates the accumulation of Amoxi-TPP-AuNPs in cell wall (A), and inside the cell (B) of *E. coli,* as shown by red arrows.

Figure 11 shows the TEM of *E. coli* after being exposed to 6 µg mL^−1^ of Amoxi-TPP-AuNPs. The Amoxi-TPP-AuNP conjugate appeared in the cell walls and inside the cells of bacteria. As a result (Figure 10 and Figure 11), the antibacterial efficacy of Amoxi-TPP-AuNPs depended on the quantity of Amoxi-TPP-AuNPs inside bacterial cells and the fast release of amoxicillin molecules from Amoxi-TPP-AuNPs [15], while amoxicillin alone was unaffected by β-lactamases and cannot enter bacterial cells.

### 3.11. Killing Rate of Amoxi-TPP-AuNPs

**Figure 12:**
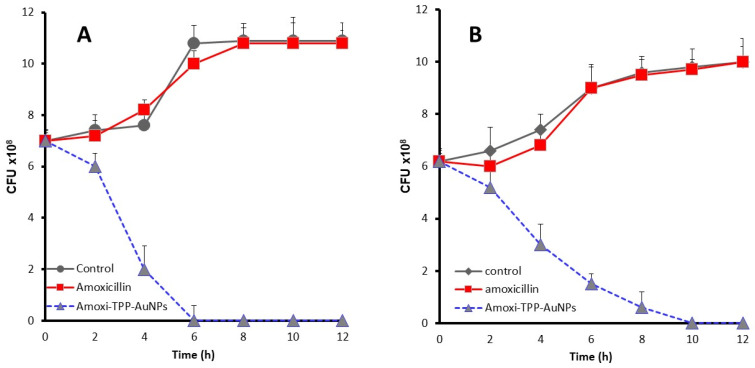
Time kill assay of *S. aureus* (MRSA) (**A**) and *E. coli* (**B**) treated with amoxicillin and Amoxi-TPP-AuNPs, compared to control amoxicillin free culture. Amoxi-TPP-AuNPs at a concentration of 8 µg/mL inhibited viable bacterial growth within 6 and 10 h, respectively.

The *S. aureus* (MRSA) and *E. coli* isolates were cultured with Amoxi-TPP-AuNPs, and samples were taken at different time intervals to count the viable bacteria; this was done to monitor the bacterial viability and death rate. Figure 12 illustrates how Amoxi-TPP-AuNPs quickly eradicate amoxicillin-resistant bacteria. Amoxi-TPP-AuNPs drastically reduced the *S. aureus* (MRSA) and *E. coli* viable count after 4 h, and the bacterial death was complete by 12 h (Figure 12A,B), similar to how Amoxi-TPP-AuNPs eliminated *S. aureus* (MRSA) and *E. coli* over the first four hours of incubation, with a discernible reduction in the viable bacterial count. Amoxi-TPP-AuNPs fully eliminated all viable bacteria after six and ten hours, respectively.

## 4. Discussion

The prevalence of bacteria that are multi-antibiotic resistant and frequently responsible for a wide range of community- or hospital-acquired illnesses represents one of the major therapeutic hurdles in healthcare-associated infections. The conjugation of amoxicillin with AuNPs using a TPP bond improves the antibiotic’s binding to AuNPs to build a unique nanodrug with a greater efficiency. Herein, we aimed to create an active nano-antibiotic employing a drug delivery system. Researchers are attempting to discover effective alternative therapeutic methods for the treatment of other resistant illnesses caused by MDR-MRSA and MDR *E. coli*. These isolates were isolated and identified using morphological, biochemical, and 16S rRNA analyses. The etiological bacterium most frequently found in urine is *E. coli*, which is also regarded as the most common cause of urinary tract infections. Skin infections, pneumonia, soft tissue infections, septic arthritis, endovascular infections, endocarditis, osteomyelitis infections from foreign substances, and sepsis are all strongly linked to *S. aureus*. Although staphylococcal bacteremia is usually associated with *S. aureus* isolation from urine samples, *S. aureus* is an uncommon cause of urinary tract infections in the general population. The PBP2a protein is produced by the mecA gene of the chromosomal cassette mec (SCCmec)-encoded MRSA bacterium. It protects the bacterial cell walls of MRSA bacteria against the effects of β-lactam antibiotics. This results in total resistance to all β-lactam antibiotics, including semi-synthetic penicillin, according to our findings and related studies [18].

In the current work, a new Amoxi-TPP-AuNP nano-antibiotic was effectively created from AuNPs conjugated with amoxicillin utilizing TPP to create a potent novel nano-drug. SPR excitation caused the AuNP solution to turn a red color [33]. As the HAuCl_4_3H_2_O concentration was raised, the absorbance peak of the AuNPs fell, suggesting particle precipitation, aggregation, and instability. Research by Markus et al. [34] also demonstrated that AuNP production took place at lower salinity levels and that larger concentrations were observed to be widened. *J. excelsa* extract contains phenolic compounds ((4-ethynylphenyl) diphenylmethoxymethane, 4-chloro-2,6-dimethylphenol, p-vinylphenol), alcoholic compounds (Aristol-9-en-3-ol, Aspidinol, Dehydroxy-isocalamendiol, Spathulenol), esters (2-ethylbutyric acid, heptadecyl ester, Vanillic acid ethyl ester), and amines (2-(1-ethoxycarbonyl-2,2,2-trifluoro-1-pentanoy lamino-ethylamino)-4-ethyl-5-methy), accounting for the formation of AuNPs [18]. Pure Au nanocrystals often exhibit XRD pattern. UV-Vis spectroscopy was used to confirm the AuNP peak at 526–535 nm caused by SPR, whereas Amoxi-TPP-AuNPs seemed to have two peaks, with the first at 517 nm being connected to AuNPs and the second at 341 nm being associated with amoxicillin [26,35]. The net negative charge of the phosphate group of TPP on the surface of TPP-AuNPs may be readily conjugated to the protonated amino group in amoxicillin, which has a positive charge [36]. Due to the tiny size of the AuNPs, these nanoparticles have a larger surface area for maximal drug loading and increased accessibility to exact targets. To combat diseases caused by resistant bacteria, various drug-conjugated nanoparticles have recently been developed [36]. As such, a lower amount of the nano-antibiotic would be needed for therapeutic use, since Amox-TPP-AuNPs had a good conjugating efficiency and did not squander it during the synthesis process [21]. As a result, the amoxicillin release from Amox-TPP-AuNPs reached 76% in the first 12 h and over 90% in the next 72 h. This behavior can be attributed to the particle size effect. The higher concentration of amoxicillin combined with the same value of small-sized nanoparticles resulted in a greater likelihood of amoxicillin molecule repulsion and faster diffusion over the dialysis membrane. As a result, the size of AuNPs allows for control over the pace of amoxicillin release [25]. When compared to free antibiotics, free AuNPs, or nano-antibiotics prepared without linkage, as well as other types of nano-antibiotics prepared using other linkers, the Amoxi-TPP-AuNPs showed a significant increase in anti-bacterial activity and were more effective against MDR bacteria. The active Amoxi-TPP-AuNPs had a MIC ranging from 3.6 to 8 g mL^−1^. When compared to other nano-antibiotics synthesized without a linker with a MIC of 300 g mL^−1^, as synthesized in earlier investigations, the antibacterial activity of Amoxi-TPP-AuNPs was enhanced 37.5 to 83 fold [37]. The Amoxi-TPP-AuNPs showed a significant improvement in antibacterial activity in conjunction with a decrease in AuNP particle size [25]. AuNPs have been exploited as drug delivery agents in several investigations [38] because of their distinctive physiochemical characteristics, including biocompatibility and non-cytotoxicity. On the other hand, AuNPs exhibited toxic effect on MCF-7 cancer cells, as recorded in our previous study [21]. When AuNPs interact electrostatically with the membrane, the membrane’s integrity is compromised [39]. By producing holes in the membrane, they can lead to intracellular component leakage. By inhibiting transcription and replication, AuNPs can attach to DNA. ROS, which are necessary for cell death, were also produced as a result of interactions between AuNPs. They change the membrane potential and lessen the activity of ATP synthase, which inhibits a number of metabolic processes. Additionally, we assessed the capacity of this nano-antibiotic to overcome penicillinase (β-lactamase) producing bacteria and neutralize bacteria that cause serious infections. Amoxi-TPP-AuNPs were shown to have the ability to remain stable in the presence of these enzymes [40]. Amoxicillin normally inhibits mucopeptide production in the bacterial cell wall [36], resulting in cell membrane rupture caused by Amoxi-TPP-AuNPs. As it enters bacteria, this medication interacts with phosphorus in DNA or RNA, interfering with all processes that regulate the membrane, including protein synthesis. Since they include numerous active groups, including the amine group of both the amoxicillin and the amine group of the AuNPs, which are joined together via the TPP linker, Amoxi-TPP-AuNPs are remarkably effective [41].

## 5. Conclusions

This study investigated the potential of TPP-AuNPs as a delivery system for amoxicillin to treat MDR bacterial infections. The surface of the sodium tri-polyphosphate-conjugated AuNPs appeared to combine well with amoxicillin. The AuNPs demonstrated good loading ability. The results of an in vitro amoxicillin release experiment showed long-lasting 72-h amoxicillin release from AuNP surfaces. Amoxi-TPP-AuNPs revealed possible antibacterial efficacy against several MDR isolates, while non-loaded AuNPs did not exhibit any antibacterial properties. Future research has been recommended to focus on the utilization of these nano-carriers for in vivo/clinical applications, i.e., delivering amoxicillin and the development of other antibiotics.

## Figures and Tables

**Table 1:** Morphological and biochemical characteristics of bacteria isolated from different sources.

Characteristics	ESC Isolates	STA Isolates
Gram’s stain	−	+
Cocci	−	+
Rods	+	−
Urease	−	+
Nitrate Reduction	+	+
Motility	+	−
Catalase test	+	+
Coagulase test	−	+
Oxidase test	−	−
Indole test	+	−
Methyl red	+	+
Voges-proskaure	−	+
Citrate	−	+
H_2_S Production	−	−
Fermentation		
Glucose	+	+
Lactose	+	+
Mannitol	+	+
Maltose	−	+
Probable bacteria	*E. coli*	*S. aureus*

**Table 2:** Resistance patterns of *E. coli and S. aureus* isolates from different sources.

Bacterial Isolates	Antibiotic Resistance Pattern	No. of Antibiotics
ESC1	AMP, AMC, CLR, CIP, LVX, SXT, LEX, CLI, CFR, CRO,	10
ESC2	AMP, AMC, CLR, CIP, LVX, SXT, LEX, CLI, CFR, AZM	10
ESC3	AMP, AMC, CEC, FOX, FEP, CXM, CAZ, CRO, CTX, CIP, LVX, DO, SXT, LEX, CLR, AZM, CLI, FUX, **CFR**	19
ESC4	AMP, AMC, CLR, DO, LEX, SXT, CLI, CFR, GEM	9
ESC5	AMP, AMC, CEC, FOX, FEP, CXM, CAZ, CRO, CTX, CIP, LVX, DO, SXT, LEX, CLR, AZM, CLI, CFR, AK	19
ESC6	AMP, AMC, CLR, DO, LEX, AZM, CRO, FUX	8
ESC7	AMP, AMC, CEC, FEP, CLR, CXM, CAZ, CRO, CTX, CIP, LVX, DO, SXT, LEX, FUX	15
ESC8	AMP, AMC, CLR, CIP, CAZ, SXT, LEX, CLI, CFR, AZM	10
^#^ STA6	CEC, PEN, ERY, FOX	4
ESC9	AMP, AMC, CLR, CIP, LVX, SXT, CEC, FEP, CXM, CRO, CTX	11
ESC10	AMP, AMC, CEC, FEP, FOX, CXM, CAZ, CRO, CTX, DO, SXT	11
ESC11	AMP, AMC, CEC, FEP, FOX, CXM, CAZ, CRO, CTX, DO, GEM	11
^#^ STA1	AMP, AMC, ERY, PEN, DO	5
^#^ STA2	AMP, PEN, ERY, CLR, AZM	5
* STA3	AMP, AMC, CEC, FOX, CXM, CIP, LVX, GEN, MEM, OXA, PEN, LEX, FEP, CRO, CFR	15
* STA4	AMP, AMC, CEC, FOX, CXM, CIP, LVX, GEN, OXA, PEN, LEX, FEP, CRO, CFR	14
* STA5	AMP, AMC, CEC, FOX, CXM, MEM, OXA, PEN, LEX, CRO, CFR	11
* STA7	AMP, AMC, CEC, FOX, CXM, MEM, OXA, PEN, ERY	9
* STA8	AK, AMC AZM, CEC, FOX, CXM, CIP, LVX, OXA, PEN, CLR, CLI, ERY	13
* STA9	AMC, CEC, PEN, ERY, GEM, OXA	6

AK, Amikacin; AMC, Amoxy/Clavulanic acid; CEC, Cefaclor; FEP, Cefepime; CTX, Cefotaxime; FOX, Cefoxitin; CAZ, Ceftazidime; CRO, Ceftriaxone; CXM, Cefuroxime; DO, Doxycycline/HCL; CIP, Ciprofloxacin; LVX, Levofloxacin; CLR, Clarithromycin; CLI, Clindamycin; AMP, Ampicillin; LEX, Cefalexin; CFR, Cefadroxil; FEP, Cefepim; MEM, Meropenem; SXT, Sulpha/Trimethoprim; FUX, Fluxocillin; GEM, Gentamicin; IPM, Imipenem; NTF, Nitrofurantoin; AZM, Azithromycin; PEN, Penicillin; OXA, Oxacillin; ERY, Erythromycin; VAN, Vancomycin. * MRSA: Methicillin-resistant *Staphylococcus aureus, ^#^* MSSA: Methicillin-susceptible *Staphylococcus aureus*.

**Table 3:** Zone of inhibition (ZIN) and MIC and MBC values of amoxicillin and Amoxi-TPP-AuNPs (15.99–24.71 nm) against MDR pathogenic *E. coli* and *S. aureus*.

Isolate	Amoxicillin (µg/mL)		Amoxacillin-TPP-AuNP (µg/mL)		
	^1^ ZIN (mm)	^2^ MIC	^1^ ZIN (mm)	^2^ MIC	^3^ MBC
ESC1	^4^ ND	108	32	4	16
ESC2	ND	114	34	8	16
ESC3	ND	108	37	8	32
ESC4	ND	108	32	4	16
ESC5	ND	114	34	8	32
ESC6	ND	108	24	8	12
ESC7	ND	102	32	8	16
ESC8	ND	102	34	4	16
ESC9	ND	108	34	4	16
ESC10	ND	102	34	8	16
ESC11	ND	96	37	4	12
* STA3	ND	114	20	8	32
* STA4	ND	108	22	8	32
* STA5	ND	114	24	4	16
* STA7	ND	114	26	3.6	16
* STA8	ND	108	23	8	32
* STA9	ND	108	26	3.6	12

^1^ Zone of inhibition by well diffusion assay, ^2^ The minimum inhibitory concentration, ^3^ Minimum bactericidal concentration, ^4^ Not detected and * MRSA.

**Table 4:** MIC values of amoxicillin and Amoxi-TPP-AuNPs (15.99–24.71 nm or 50.34–100.45 nm) against MDR pathogenic *E. coli* and *S. aureus*.

	MIC (µg/mL)		
	Amoxicillin	Amoxi-TPPi-AuNPs (15.99–24.71 nm)	Amoxi-TPPi-AuNPs (50.34–100.45 nm)
ESC1	ND	4	8
ESC2	ND	8	16
ESC3	ND	8	16
ESC4	ND	4	8
ESC5	ND	8	16
ESC6	ND	8	16
ESC7	ND	8	16
ESC8	ND	4	8
ESC9	ND	4	8
ESC10	ND	8	16
ESC11	ND	4	8
* STA3	ND	8	16
* STA4	ND	8	16
* STA5	ND	4	8
* STA7	ND	3.6	8
* STA8	ND	8	16
* STA9	ND	3.6	8

ND = Not detected and * MRSA.

## Data Availability

Data will be made available upon request from the corresponding author.
